# Grapevine Phyllosphere Community Analysis in Response to Elicitor Application against Powdery Mildew

**DOI:** 10.3390/microorganisms7120662

**Published:** 2019-12-07

**Authors:** Luca Nerva, Chiara Pagliarani, Massimo Pugliese, Matteo Monchiero, Solène Gonthier, Maria Lodovica Gullino, Giorgio Gambino, Walter Chitarra

**Affiliations:** 1 Research Centre for Viticulture and Enology, Council for Agricultural Research and Economics (CREA-VE), Via XXVIII Aprile 26, 31015 Conegliano, Italy; 2 Institute for Sustainable Plant Protection, National Research Council (IPSP-CNR), Strada delle Cacce 73, 10135 Torino, Italy; chiara.pagliarani@ipsp.cnr.it (C.P.); solene.gonthier@insa-lyon.fr (S.G.); giorgio.gambino@ipsp.cnr.it (G.G.); 3 Centre of Competence for the Innovation in the Agro-Environmental Sector (AGROINNOVA), University of Torino, Largo Paolo Braccini 2, 10095 Grugliasco (TO), Italy; massimo.pugliese@unito.it (M.P.); marialodovica.gullino@unito.it (M.L.G.); 4 Department of Agricultural, Forest and Food Sciences (DISAFA), University of Torino, Largo Paolo Braccini 2, 10095 Grugliasco (TO), Italy; 5 Ant-Net S.r.l., Via Livorno 60, 10140 Torino, Italy; m.monchiero@alice.it; 6 Biocomputing and Modelling Department, National Institute of Applied Sciences, INSA Lyon, 69621 Villeurbanne cedex, France

**Keywords:** *Erysiphe necator*, fungal community, microorganisms, organic compounds, phyllosphere, resistance induction, virome, *Vitis vinifera*

## Abstract

The reduction of antimicrobial treatments and mainly the application of environmentally friendly compounds, such as resistance elicitors, is an impelling challenge to undertake more sustainable agriculture. We performed this research to study the effectiveness of non-conventional compounds in reducing leaf fungal attack and to investigate whether they influence the grape phyllosphere. Pathogenicity tests were conducted on potted *Vitis vinifera* “Nebbiolo” and “Moscato” cultivars infected with the powdery mildew agent (*Erysiphe necator*) and treated with three elicitors. Differences in the foliar microbial community were then evaluated by community-level physiological profiling by using Biolog^TM^ EcoPlates, high throughput sequencing of the Internal Transcribed Spacer (ITS) region, and RNA sequencing for the viral community. In both cultivars, all products were effective as they significantly reduced pathogen development. EcoPlate analysis and ITS sequencing showed that the microbial communities were not influenced by the alternative compound application, confirming their specific activity as plant defense elicitors. Nevertheless, “Moscato” plants were less susceptible to the disease and presented different phyllosphere composition, resulting in a richer viral community, when compared with the “Nebbiolo” plants. The observed effect on microbial communities pointed to the existence of distinct genotype-specific defense mechanisms independently of the elicitor application.

## 1. Introduction

Grapevine is one of the most economically important crops worldwide, and several pathogens are known to affect the yield and quality of the final product. This results in the extensive use of pesticides and fungicides. The viticulture sector is considered one of the most treated cropping systems [1] as, over the growing season, an intensive fungicide schedule is commonly adopted to control the spread of pathogens in the vineyard (from about 12 to 30 treatments are applied per season) [2]. Based on official data retrieved from the European Union website [3], over the last years, the use of pesticides has progressively risen, especially in the case of fungicides, which account for about 60% of the total pesticide volume applied in EU. These increases are thought to be due to climate change effects, such as high temperatures and high humidity conditions occurring during the growing season [4,5]. 

Among the most important fungal pathogens affecting grapevine, powdery mildew is a serious phytosanitary threat in the major European wine-growing regions, including Northern Italy, where it causes severe crop and quality losses if no control measures are carried out. Powdery mildew is a ubiquitous disease, and its causal agent, *Erysiphe necator* (syn. *Uncinula necator*), is a polycyclic fungus able to infect all the plant green tissues by producing a grey-white mycelium [6]. Usually, powdery mildew and other diseases are mainly managed by chemical fungicides, including copper and sulfur, which are also widespread in organic farming. However, over the past few years, researchers and local governments have worked to reduce the application of conventional fungicides in order to minimize the impact on both the environment and human health [7]. The European Commission (EC) has applied serious restrictions on the number of chemical treatments (Directive 2009/128/EC). More recently, copper, which is still considered the only effective product in organic viticulture, has further been constrained by the EC (Regulation 2002/473/EC and Commission Implementing Regulation 2018/1981). 

To date, numerous efforts have been made to lower and/or replace the input of conventional chemical fungicides in viticulture by combining different approaches. For example, specific agronomic techniques have been adopted to reduce pathogen inoculation and avoid microclimate conditions favorable for fungal infections. Grapevine varieties resistant or tolerant to powdery and downy mildews have been obtained, although the wines produced by these hybrid genotypes are not satisfactory for consumers (e.g., presence of undesired off-flavors) or not compatible with European Union schemes of geographical indications and traditional specialties [8,9]. Additionally, it has become increasingly important to develop more environmentally friendly products, such as biofungicides, resistance inducers, and elicitors. Specifically, elicitors of plant defense responses are natural or natural-derived molecules (widely distributed in their chemical nature) able to mimic the presence of either a pathogen or molecules produced by plants in response to pathogen attack [10,11]. These naturally-derived molecules are generally assumed to be less hazardous for the ecosystem since they are easily converted into the organic matter [12,13]. Examples of these are laminarin, potassium phosphonate, and acibenzolar-S-methyl. Laminarin is extracted from the brown algae *Laminaria digitata*, known to stimulate defense responses in plants against downy and powdery mildews and grey mold [14,15,16,17,18]. Potassium phosphonates have been only recently registered in Europe as suitable pesticides for grapevine, although their effect on downy mildew has been known since the year 2000 [19,20]. The effects of potassium phosphonates (which are similar to potassium phosphites) are still not completely clear. Available information indicates they have a double mode of action, as they directly inhibit fungal colonization and penetration from one side and stimulate plant defense responses on the other [21,22]. Acibenzolar-S-methyl is a benzothiadiazole (BTH) that mimics pathogen-host interaction, thus triggering systemic acquired resistance in plants [23,24]. 

Plants are commonly colonized by a rich diversity of microbes, represented by bacteria, fungi, and viruses, which are able to affect growth and productivity yields through several physiological mechanisms not yet fully understood. Only in the last few years, researchers started to study the bacteria associated with the phyllosphere in relation to changes of specific plant traits, such as photosynthetic rates, leaf temperature, and plant defense processes [25,26,27]. In addition, the importance of leaf microbial diversity as a factor influencing ecosystem productivity and functional relationships has already been demonstrated [28]. Consequently, plants and the interacting microorganisms have begun to be viewed as a single unique entity, referred to as holobiont [29,30]. Several virus, bacterial, and fungal species commonly constitute the phyllosphere microbial community, and many of these are unculturable. These leaf-inhabiting microbes are influenced by such limited environmental conditions (e.g., continuous fluctuations in water and nutrient availability, atmospheric conditions), as well as upon exposure to biotic and abiotic stresses, which can alter their abundance and composition [31,32,33,34,35]. This represents a further level of complexity that explains why the interplay between leaves and their inhabiting microbial communities is only partially understood today [31,36,37]. For instance, indigenous leaf microorganisms can represent a barrier against pathogens, as some of them can act as biological control agents [35] by stimulating the production of antimicrobial compounds, as well as by competition or parasitism strategies [38], or by triggering the plant innate immunity system [39]. 

In grapevine, the role and composition of leaf microbial communities were largely ignored until recently, when the concept of microbial *terroir* started to spread [40,41]. For example, the grapevine can potentially host more than 80 different viral species, of which only about half are recognized as agents of economic relevant diseases [42,43,44]. For most of these viral entities, the effects and metabolic modifications caused in grapevine are still unknown. However, mutualistic interactions have been reported, in which some viruses conferred drought tolerance and increased resistance to downy mildew (*Plasmopara viticola*) [30,43,44]. Thus, grapevine-virus interactions can be studied not only according to classical host-pathogen relationships but also by considering grapevine and its viruses as a single micro-ecosystem able to adapt to the surrounding environment and to influence plant responses, including those to fungi and fungicides. In light of this, researches were conducted to correlate genotype and environmental interaction in order to link the microbial ecology of different grapevine tissues to the presence of specific phenotypic traits [45,46,47]. In parallel, other studies reported insights into the impact of chemical and biological treatments against leaf fungal pathogens on grape foliar microbial communities [43,44]. Nevertheless, information on this topic is still limited, and available literature only takes into account the influence of treatments against downy mildew analyzing bacterial diversity. 

In particular, to the best of our knowledge, studies highlighting community changes in leaf fungal (considering both ecto- and endophytes) and viral species in response to the application of antifungal compounds are still limited. 

In the present paper, we investigated the effectiveness of alternative environmentally friendly products against powdery mildew spread, and we questioned how they influence the phyllosphere communities by making comparisons between two susceptible *Vitis vinifera* cultivars—“Nebbiolo” and “Moscato”.

## 2. Materials and Methods

### 2.1. Plant Material and Experimental Set-Up

Two consecutive experimental trials were carried out from August to September 2017 (Table 1 and Table 2) at the University of Turin Agroinnova Competence Center (Grugliasco (TO), Northwest of Italy, (GPS: 45° 03’ 57.8” N, 7° 35’ 29.5” E)), working on one-year-old potted grapevines (*Vitis vinifera* L.) of cultivars Moscato (white grape variety) and Nebbiolo (red grape variety) grafted onto Kober 5BB rootstock and maintained in an open-air environment. Plants were grown in 4 liters pots (16 × 16 × 18 cm) using a peat substrate (TS4, Turco Silvestro, Italy) and placed over a woven polypropylene geotextile mulching film under a greenhouse shade cloth, in order to protect them from leaf damaging hailstorms. The open-air system was located about 100 m far from an experimental vineyard established more than 10 years ago, where downy and powdery mildew naturally occur. Plants were artificially inoculated with a suspension of 1 × 10^5^ conidia/mL of *E. necator* according to the methodology reported in Pugliese et al., 2010 [3] and Pugliese et al., 2018 [13]. Fungal material used in this study corresponded to an isolate of *E. necator* collected from naturally infected grapevines grown in the experimental field and maintained under in vitro conditions on grapevine leaves.

Experiments were carried out on each cultivar, working on a total of 72 plants. In particular, the first trial was conducted using 40 plants (2 genotypes × 5 plants × 4 treatments (corresponding to the three commercial products and the untreated control)), while the second one was carried out on 32 plants (2 genotypes × 4 plants × 4 treatments). Each plant was used for the trials nearly 60 days after transplanting the cuttings. Culture conditions were uniform in the experimental site. Products were applied using a hand-pulled 2-stroke engine sprayer at a pressure of 15 bar. Before the first artificial inoculation with the pathogen, vines were treated twice following a 7 day-interval. After inoculation, two more treatments (at 7–8 day-interval) were carried out, followed by a second inoculation with *E. necator*. Finally, two other treatments were performed before the evaluation of disease development. The plants used as CTRL were inoculated twice with *E. necator,* without applying products for the control of the pathogen before and after the inoculation (Table 2). Disease severity (% of leaf surface affected) and incidence (% of affected leaves) were evaluated on all plants of the two consecutive trials by checking the whole plant canopy three days after the last treatment, at the onset of symptoms. 

At the end of the experiment, three biological replicates were obtained for each treatment. Each biological replicate was formed by pooling six asymptomatic leaves randomly selected from three plants (2 leaves from each plant) in each group (Table 2). The collected samples were immediately processed for Biolog^TM^ EcoPlate analysis, and the remaining material was frozen in liquid nitrogen and stored at −80 °C until nucleic acids were extracted for the sequencing process.

Three commercial formulations were applied: Acibenzolar-S-methyl (Bion, Syngenta Crop Protection) (AcS-Mt), Potassium phosphonate (Century, BASF Agro) (K-Pho), and Laminarin (Vacciplant, Arysta Lifescience) (Lam) (Table 1). Products were used according to the manufacturer’s instructions. 

### 2.2. Microbial Functional Diversity

To analyze the treatment and genotype effect on the functional diversity of the culturable phyllosphere microbial communities, a community-level physiological profiling (CLPP) analysis was performed using Biolog EcoPlates^TM^ (96-well plates containing 31 different C lyophilized substrates) (Biolog Inc., Hayward, CA, USA) on 24 samples (2 genotypes × 4 treatments × 3 replicates). To this aim, one gram of fresh leaf sample was inserted in an extraction bag (Bioreba AG, Reinach, Switzerland) containing 9 mL of 0.8% NaCl solution and macerated using a rolling ball (RDM-50A press, Rexon). Afterward, 2 mL of extracted sample was diluted 1:10 using 0.8% NaCl solution, and 150 µL of the final extract was aliquoted to each well of the microplate following incubation at 25 °C in dark conditions. Optical density (OD) readings were performed at 590 nm in a microplate reader (Bio-Rad Laboratories, Hercules, CA, USA) after 24 h of incubation and every 24 h for 7 days (measuring time from 24 h to 168 h). 

Principal component analyses (PCA) were then performed, using the corrected OD values as the input, in order to reduce data dimensionality and highlight the weight of the main factors (genotype and/or treatment) in influencing microbial biodiversity. Subsequently, the average well color development (AWCD) was calculated for each plate (serving as biological replicate) at each time point according to [48]: AWCD = ∑ C−R/31, where C is the OD of each carbon source well, and R is the OD of the control wells. The calculated AWCD data were plotted over the incubation time for each treatment and cultivar and statistically analyzed. The normality of samples was not observed; thus, the non-parametric Kruskal-Wallis test was used to estimate significant differences. 

Then, the most two common diversity indices, the Shannon’(H’) and Simpson’s (D) indices, were determined to start from AWCD data. In brief, H’ index was computed as follows:

H’ = ∑ pi ln (pi), where pi is the color development (OD) related to each substrate in the plate and scaled to the AWCD [pi = (C−R)/AWCD [48]. H’ is minimal and equal to 0 when only one carbon substrate is used, and it is maximum when all the observations (here the OD) are equally divided by all the carbon sources. In this last case, H’ is equal to ln (1/S), where S represents the number of carbon sources [49]. According to [50], a high H’ value corresponds to high functional diversity. 

The D index was formulated as follows: D = 1−∑ pi2 [51], taking into account not only the number of carbon sources used but also their proportions. According to [49], it represents the chance for two randomly taken observations to belong to the same class. If all the substrates are used, but one more than all others, their pi will be high, therefore D will be close to 0. Conversely, if there is a good repartition among all carbon sources, all pi values will be low, and D will be close to 1. The Simpson’s diversity index represents the probability that two individuals randomly selected from a sample belong to different species, hence the greater the D-value is, the greater the sample diversity is [50]. While H’ highlights differences in terms of bacteria species growing on the plate, D shows differences in terms of carbon source use, thus revealing the biodiversity of bacteria that are using that carbon source specifically, for each biological replicate, treatment, or time point considered.

To test significant differences among samples in terms of diversity indices, a Shapiro’s test was first performed to verify the normality of each biological sample (R: function shapiro.test), and if this condition was verified, an ANOVA test was then applied (R: function ANOVA). When a significant difference was found, a Tukey’s HSD test was run to highlight significant differences among treatments (function Tukey HSD with confidence level equal to 0.95). In the case of non-normality, a Kruskal-Wallis’ rank-sum test was used (R: function Kruskal.test). 

### 2.3. DNA Isolation and Sequencing

Leaf samples were collected and immediately frozen in liquid nitrogen. For each treatment and biological replicate, total DNA was extracted from 100 mg of fresh homogenized leaf samples using a plant/fungi DNA isolation kit (Norgen Biotech Corp., Thorold, ON, Canada) and following the manufacturer’s instructions. Total DNA was quantified using a NanoDrop 2000 spectrophotometer (Thermo Fisher Scientific, Waltham, MA, USA), and DNA integrity was inspected running the extracted samples on a 1% agarose electrophoretic gel.

Primers for the amplification of the fungal ITS3-ITS4 were used to amplify the highly variable spacers ITS2 of the rDNA fungal operon [52,53], and MiSeq Illumina sequencing analysis was carried out by Macrogen, Inc. (Seoul, Korea). In detail, 25 cycles of PCR (55 °C annealing temperature and 30 s of extension) on 5 ng of total DNA were used to amplify the region of interest with the above mentioned specific primer pair for ITS2. Afterward, 1 µL of PCR product was run on a Bioanalyzer DNA 1000 chip (Agilent Technologies, Santa Clara, CA, USA) to verify the amplicon size (expected size of 400 bp). PCR products were cleaned and processed using a Nextera XT index kit (Illumina, San Diego, CA, USA). Libraries were then sequenced on a MiSeq Illumina apparatus according to the manufacturer’s instructions.

### 2.4. Fungal Metaphylogenomic Analyses and Taxonomic Distributions 

Raw reads obtained from the MiSeq sequencing process were cleaned of adaptors and filtered using a Macrogen Inc. in-house script based on the Illumina package bcl2fastq v1.8.4. The obtained pre-cleaned reads were first subjected to strict quality control with PrinSeq v0.20.4 [54] and then processed in Qiime 2 [55]. Retained reads were then used to identify the start and stop sites for the ITS region using the hidden Markov models (HMMs) [56]. The software was developed for the marker gene (16S or ITS) studies using operational taxonomic units (OTUs). In order to distinguish true sequences from those containing errors, sequences were sorted by abundance and then clustered in a greedy fashion at a threshold percentage of identity (97%) [56]. Trimmed sequences were then analyzed with DADA2 [57]. Variants identified in the previous steps were taxonomically classified through the dynamic UNITE database [58]. For graphic representation, only genera with an average relative abundance higher than the settled threshold (0.1%) were retained. 

### 2.5. RNA Extraction, Sequencing, and Virome Analysis

For each treatment and biological replicate, total RNA was extracted starting from 100 mg of leaf material using the Spectrum^TM^ plant total RNA kit (Merck KGaA, Darmstadt, Germany), following the manufacturer’s instruction. Total RNA yield and purity were determined using a NanoDrop 2000 spectrophotometer (Thermo Fisher Scientific, Waltham, MA USA), and integrity was checked with a 2100 Bioanalyzer (Agilent Technologies, Waldbronn, Germany). RNA libraries were then obtained using the TruSeq RNA library V2 kit and sequenced on a NovaSeq Illumina apparatus following a paired-end approach, producing an output of 30 million reads per sample (Macrogen, Inc., Seoul, Korea). As above reported, NovaSeq sequences were trimmed from adaptors and filtered using a Macrogen Inc. in-house script based on the Illumina package bcl2fastq v1.8.4.

To detect viruses, viroids, and phytoplasmas, a custom dataset was built by retrieving available sequences from the NCBI database. Raw reads resulting from the NovaSeq sequencing were trimmed from adaptors and quality filtered using Trimmomatic [59], then mapped against the custom database using BWA and SAM tools [60,61]. Virus and viroid infections were further validated using previously described PCR end-point protocols [62,63,64]. 

### 2.6. Statistical Analyses

Analysis of variance (ANOVA) was applied to detect statistically significant differences in disease and severity index data. When ANOVA indicated that either genotype (G: “Nebbiolo”, “Moscato”) or treatment (T: CTRL, AcS-Mt, K-Pho, Lam) or their interaction (G × T) was significant, mean separation was performed by Tukey’s honestly significant difference (HSD) (*p* ≤ 0.05) using the SPSS statistical software package (v. 23.0; SPSS Inc., Cary, NC, USA). Genotype (G) main effects were compared using the Student’s *t*-test. The same approach was followed to assess significant differences in *Erysiphe* OTUs abundance between genotypes.

To analyze Biolog EcoPlates, the principal component analysis (PCA) was performed using the R (Version 3.4.4) software [65] with the packages ade4 [66] and adegraphics [67] to extract the main factors influenced by the applied treatments. A Shapiro’s test was first used to verify the normality distribution of each sample. Diversity index data were shown as mean values, and an ANOVA analysis followed by Tukey’s HSD test was applied to separate the means when significant (*p* ≤ 0.05). In the case of non-normality, a Kruskal-Wallis’ rank-sum test was used. Statistical computations were performed using the SPSS software package.

Statistical analysis for microbiome data was performed with R (Version 3.4.4) using phyloseq (version 1.24.0) to import, store, and analyze data [68]. The official extension phyloseq to_deseq2 was used to convert data from phyloseq to DESeq2 data set [69]. The 24 communities obtained from the ITS analysis (2 genotypes × 4 treatments × 3 replicates) were used to evaluate beta-diversity deriving from the Bray-Curtis distance matrix (complete dataset were considered at the OTU level clustered with a cut-off threshold of 97% identity). The matrix was further used as input to run a non-parametric multivariate analysis (PERMANOVA) (*p*-value were corrected with sequential Bonferroni significance) and a non-metric multidimensional scaling (NMDS) using the PAST software [70].

## 3. Results

### 3.1. Disease Development

**Figure 1:**
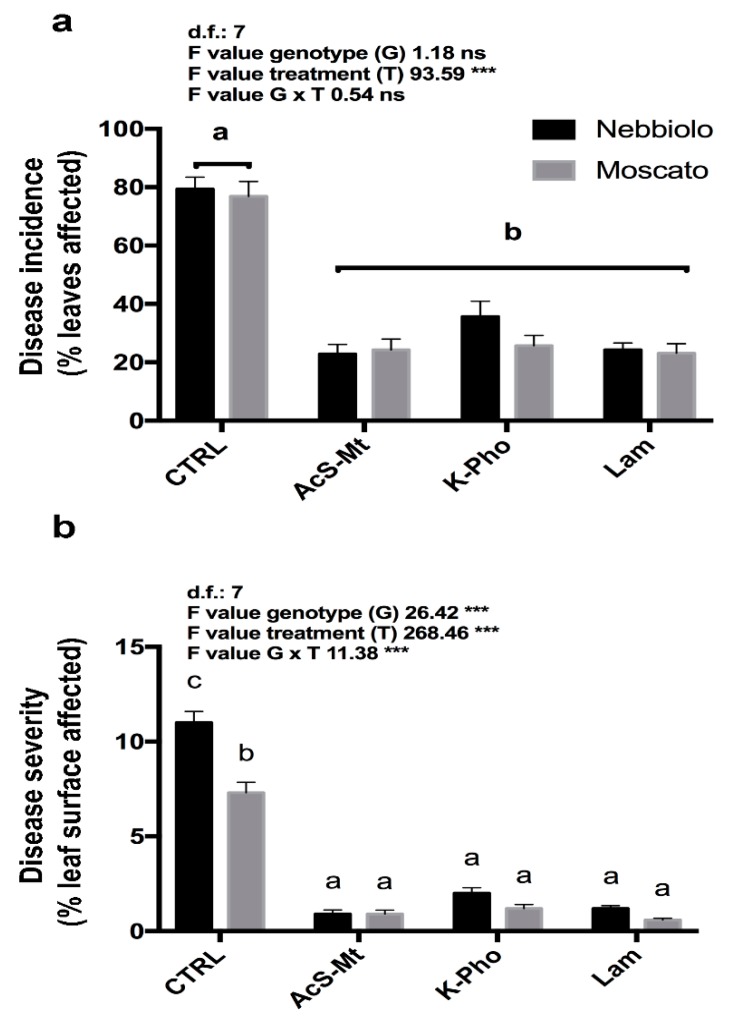
Disease incidence (**a**) and severity (**b**) of *E. necator* infection in “Moscato” and “Nebbiolo” varieties. Plants were artificially inoculated with the pathogen and untreated (CTRL) or treated with acibenzolar-S-methyl (AcS-Mt), potassium phosphonate (K-Pho), and laminarin (Lam). Lower case letters above bars indicate significant differences (*p* ≤ 0.05) as attested by Tukey’s HSD. ns denotes no significant differences; *** denotes significant differences (*p* ≤ 0.001). Genotype (G) main effects were assessed by Student’s *t*-test.

All formulations were able to efficiently reduce the disease incidence, in terms of percentage of leaves affected, and the disease severity, in terms of percentage of leaf surface affected, with an efficacy higher than 50% in both the tested cultivars (Figure 1). In detail, the analysis of variance conducted on disease incidence data showed a significant effect due to the treatment (T). For both cultivars, the application of the three products significantly reduced the disease incidence in comparison with control plants (CTRL) (Figure 1a). 

Overall, the three products also significantly reduced disease severity in both the varieties (Figure 1b). Moreover, the analysis of variance conducted on the disease severity data highlighted a highly significant effect due to genotype (G), treatment (T), and their interaction (G × T) (Figure 1b). In particular, already in the absence of treatments (CTRL), a genotype effect was observed, as in “Moscato”, the disease severity was significantly lower (about 40% less) than “Nebbiolo”.

### 3.2. Treatment Effects on Microbial Functional Diversity

The effects of the imposed treatments on the potential functionality of culturable phyllosphere microbial communities were analyzed by community-level physiological profile (CLPP) technique using Biolog EcoPlates^TM^. Although this assay was commonly applied in the analysis of soil substrates [51,71], it was already successfully used to profile the functional diversity of heterotrophic microbial communities present in grapevine leaves [72]. 

**Figure 2:**
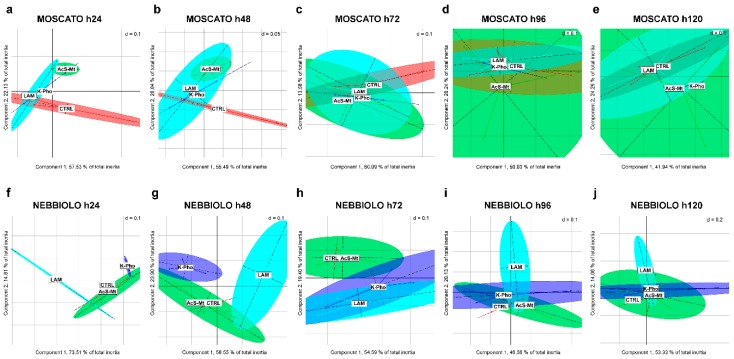
Functional diversity of phyllosphere bacterial communities analyzed by Biolog^TM^ EcoPlate. PCA analysis was carried out on all biological replicates of “Moscato” (**a**–**e**) and “Nebbiolo” (**f**–**j**) plants using absorbance data (OD_590_) over a time course of five days (from h24 to h120). Data were grouped by treatments: inoculated untreated control (CTRL, red), acibenzolar-S-methyl (AcS-Mt, green), potassium phosphonate (K-Pho, violet), and laminarin (Lam, light blue). Ellipses represent the 95% confidence intervals.

A PCA analysis was first performed on absorbance values collected for each Biolog EcoPlate over a time course of 5 days from plate incubation (h24 to h120), keeping the two cultivars separate. Differences in terms of microbial potential activity, linked to the presence of a specific treatment, were observed at early time points (Figure 2). More in detail, in “Moscato”, at h24 and h48, AcS-Mt clustered separately from CTRL, while the two unconventional treatments, K-Pho and Lam, grouped along the whole time course (h24 to h120). However, after 3 days of incubation (h72), all the 95% confidence intervals overlapped (Figure 2c,h). A different trend was noticed for “Nebbiolo”. At h24 and h48, the controls clustered together with AcS-Mt, while the other treatments formed two separate groups. Then, at h72, while AcS-Mt still grouped with the control, Lam and K-Pho partially overlapped, and at h96 and h120, not all treatments were distinguishable anymore, as also happened in “Moscato” (Figure 2i,j). However, it must be noticed that, for both cultivars, a complete separation related to a specific treatment was not evident in the clusters generated by PCA. 

Therefore, in order to decipher whether the genotype effect weighted more than the treatment in influencing microbial activity, further PCA analyses were carried out. First, the two cultivars were directly compared at each time point, analyzing absorbance values from all treatments together (Figure S1). The obtained results evidenced that the effect of genotype was indeed much stronger than that exerted by fungicide treatments. Indeed, since early in the time course, clusters ascribed to different “Moscato” samples overlapped together and could be easily distinguished from those related to “Nebbiolo”. Separation among cultivars was particularly evident, starting from 72 h after plate incubation (Figure S1). Finally, one last PCA was run, in which each treatment was extracted to better display differential response due to the genotype effect (Figure S2). The results attested to a distinction between the two cultivars that were always present when absorbance values obtained from plates of the same treatment were analyzed (Figure S2). In the case of K-Pho treatment, “Moscato” and “Nebbiolo” completely differed only at h120 (Figure S2c), whereas for Lam, microbial functional diversity was already detectable at h24 and h48 (Figure S2d). Overall, after 3 days (h72) of plate incubation, the major variations in phyllosphere microbial communities in response to treatments were observed, and for this reason, the analysis of microbial diversity indices (Simpson’ (D) and Shannon’s (H’) indices, see below) was conducted using the 72 h time point as the reference. 

The analysis of the average well color development (AWCD) of all carbon substrates was plotted over the incubation time for each treatment and cultivar (Figure S3). Independently of the analyzed genotype, no significant differences were observed when AWCD values obtained for the same treatment were compared over time (Figure S3). 

Finally, in order to reveal the biodiversity of culturable bacteria using a specific carbon source in each condition, Simpson’s (D) and Shannon’s (H’) indices were calculated using the AWCD values previously obtained and following already published methods [49,50,51,73]. Given that the major variations in phyllosphere microbial communities in response to treatment/genotype were observed at 72 h, only AWCD data at 72 h of plate incubation (see above) were used to calculate Shannon–Wiener (H’) and Simpson (D) diversity indices. No significant differences among samples were observed from the analysis of H’ and D indices in both “Nebbiolo” and “Moscato” (Table 3). 

### 3.3. Treatment Effects on Fungal Community Diversity and Composition

Phyllosphere fungal communities were investigated by high throughput sequencing of ITS regions (NCBI accession numbers from SRR8279763 to SRR8279786). After read cleaning, the resulting MiSeq sequences ranged from 290 to 302 bp, and the total number of reads per sample ranged from 159260 to 214642 counts, as summarized in Table S1. The species accumulation curve tended to saturation when the number of reads increased, thus indicating that the sequencing depth was sufficient (Figure S4).

**Figure 3:**
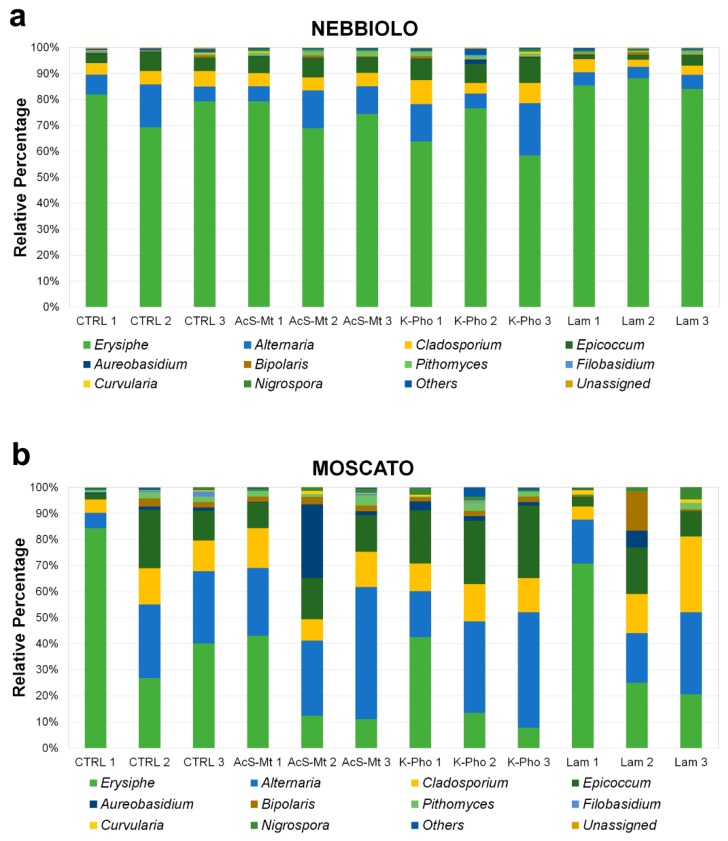
Comparison of “Nebbiolo” (**a**) and “Moscato” (**b**) fungal phyllosphere community structures among treatments based on ITS amplicon sequencing at the end of the experiment. The three biological replicates are shown separately. The taxonomic comparison was made at the genus level. Inoculated untreated control (CTRL), acibenzolar-S-methyl (AcS-Mt), potassium phosphonate (K-Pho), and laminarin (Lam). NE: “Nebbiolo”, MO: “Moscato”.

To analyze fungal community diversity, we focused on the differences in quantity amounts and profile distribution at the fungal genus level in relation to diverse treatments or cultivars (Figure 3). To better magnify differences, data were reduced, excluding genera with an average relative abundance below 0.1% (Figure 3). No significant differences in either community diversity composition or genus abundance were detected by comparing the control and treated samples within the same genotype. The average abundance of *Erysiphe* genera in ‘Nebbiolo’ samples accounted for 67% (NE K-Pho) up to 86% (NE Lam) of sequenced reads, whereas, in “Moscato”, *Erysiphe* assigned reads ranged from 21% (MO K-Pho) up to 50% (MO CTRL). The second most abundant genus was *Alternaria*, which accounted for 1% to 13% and 20% to 35% in “Nebbiolo” and “Moscato”, respectively, followed by *Cladosporium* (3% to 7% in “Nebbiolo” and 10% to 16% in “Moscato”) and *Epicoccum* (2% to 8% in “Nebbiolo” samples and 10% to 24% in “Moscato”) (Figure 3 and Table S2). Complete information about the identified OTUs can be found in Tables S3 and S4 for “Moscato” and “Nebbiolo”, respectively. 

**Figure 4:**
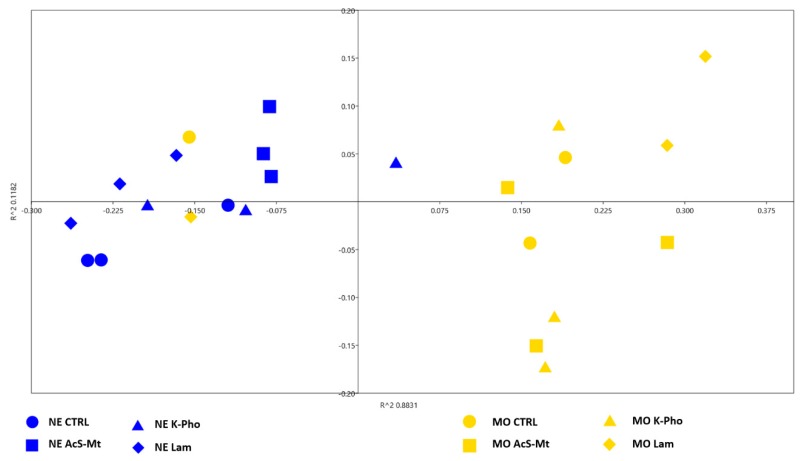
Bi-dimensional scaling of fungal microbial community data. Reduced representation of ITS sequencing data obtained by applying the NMDS (non-metric multidimensional scaling) algorithm, based on Bray-Curtis distance matrices. Inoculated untreated control (CTRL), acibenzolar-S-methyl (AcS-Mt), potassium phosphonate (K-Pho), and laminarin (Lam). NE: “Nebbiolo”, MO: “Moscato”.

**Figure 5:**
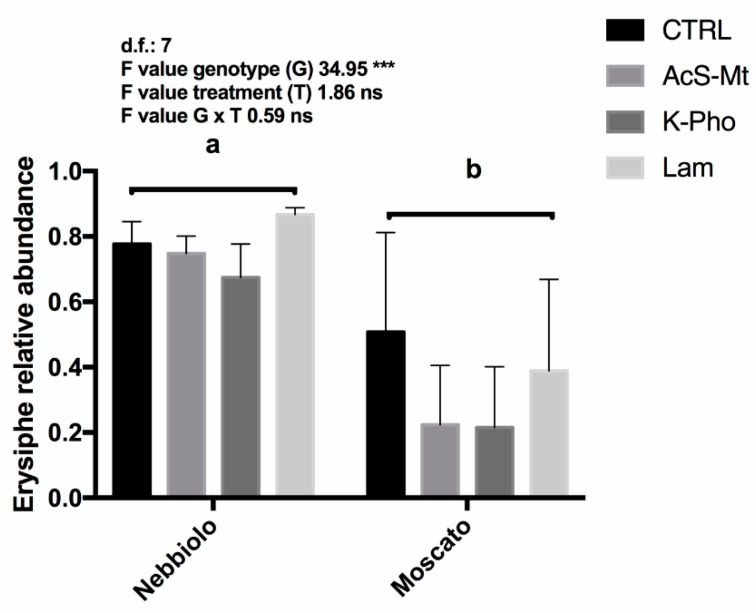
*Erysiphe* relative abundance among treatments. Plants were artificially inoculated with the pathogen and then untreated (CTRL) or treated with acibenzolar-S-methyl (AcS-Mt), potassium phosphonate (K-Pho), and laminarin (Lam). Lower case letters indicate significant differences (*p* ≤ 0.05) as attested by Tukey’s HSD. ns denotes no significant differences; *** denotes significant differences (*p* ≤ 0.001). Genotype (G) main effects were assessed by Student’s *t*-test.

Bray-Curtis matrix was used to perform PERMANOVA analyses (Tables S5–S8). The PERMANOVA results confirmed that the treatments had no significant effects on the fungal community composition, while they highlighted a highly significant effect of genotype (*p* = 0.0001). Furthermore, following bioinformatics classification, results were summarized by reducing the dataset to a bi-dimensional scale, using a Bray-Curtis distance matrix (based on OTUs), and the coordinates were plotted by corresponding non-metric multidimensional scaling (NMDS). The NMDS plot showed that, independently of treatments, samples had a tendency to cluster according to the cultivar (Figure 4). To better explain differences between the two genotypes, a DESeq2 analysis was carried out to highlight significant variations in the percentage of *Erysiphe* (*p* < 0.001) between “Moscato” and “Nebbiolo”. Moreover, the analysis of variance, conducted only on *Erysiphe* OTU abundance in “Nebbiolo” and “Moscato” samples, displayed a highly significant genotype effect (Figure 5). Finally, also, alpha diversity indices (Table S9) were calculated for each set of replicates, and no significant differences were observed. 

### 3.4. Grapevine Virome, Viroid, and Phytoplasma Analysis

A summary of the RNA-seq (NCBI accession numbers from SRR8279892 to SRR8279915) statistics and metrics is reported in Table S10. The custom dataset was built using the genome sequences of 75 viruses [74], five viroids, and three phytoplasmas, for a total of 119 reference accessions (Table S11). Although no viral symptoms were visible over the duration of experiments, 14 viruses and three viroids were detected, but their distribution was not uniform among treatments and cultivars. Interestingly, richness in viruses and viroids was higher in “Moscato” than “Nebbiolo” plants. Particularly, “Nebbiolo” vines were infected by three viruses (grapevine rupestris stem pitting associated virus, grapevine pinot gris virus, and grapevine fleck virus) and two viroids (grapevine yellow speckle viroid 1 and hop stunt viroid), whereas “Moscato” plants were infected by 13 viruses and three viroids (Table 4). The RNA-seq data were also experimentally validated by using dedicated multiplex end-point PCR protocols for virus and viroid detection, and sequencing results were successfully confirmed (Figure S5). No phytoplasmas were detected in any of the analyzed samples. 

## 4. Discussion

Over a long time, the grapevine has been selected in order to maintain or improve different phenotypic traits linked to quality, productivity, or biotic/abiotic adaptation strategies. This selection led to many different clonal varieties (i.e., genotype). During the last few years, it was uncovered that, besides genotype characteristics, commensal microorganisms harbored by the plant play key roles in the modulation of grape quality features [40,75,76] and plant health [77]. All these findings have highlighted the importance of in-depth studies on the complex relationships existing between plants and their native associated microbiota. New knowledge on this matter would be crucial to orient and improve sustainable viticultural practices, as well as to develop new control strategies [27,28,32]. 

In response to the ongoing global climate change, and considering the increasing need to adopt environmentally friendly pesticides, many products have been tested to reduce the massive chemical inputs applied against grape pathogens [78]. Here, we observed, as previously demonstrated by other works [18,21,23], that plant elicitors could be adopted as alternative strategies to effectively control powdery mildew.

To date, only a few works have attempted to elucidate the effect(s) of the application of eco-friendly compounds on grape-associated microflora communities, focusing on those residing on the leaf surface [43,44]. In this study, the impact of elicitors was tested by focusing on community changes occurring at the level of indigenous leaf microbial endo- and ectophyte populations. We combined RNA and DNA high throughput sequencing approaches to identify viral and fungal communities by comparing leaf samples of two different *Vitis vinifera* cultivars, the red-grape “Nebbiolo” and the white-grape “Moscato”, both largely cultivated in North-western Italy. Microbial functional diversity was also inspected by means of the Biolog EcoPlate^TM^ technique. Integration of these data with disease indices revealed that, besides an evident genotype effect, AcS-Mt, K-Pho, and Lam successfully acted against powdery mildew spread, consistent with the previous observations [18,24,79]. Since our experimental set-up was based on an environmentally friendly protocol, these findings confirmed the effectiveness of the tested products and opened the venue for their employment in disease management programs in viticulture. 

The microbial community-level physiological profiling (CLPP) analysis, we adopted, represents a powerful and quick tool that has been successfully used to compare the functional biodiversity of culturable microbial communities in soils [73] and phyllosphere [32], or to evaluate the toxicological impacts of pollutants [80]. Although this technique could not provide information on the microbial taxonomic structure and its composition, results evidenced that the metabolic activities of the culturable indigenous leaf microbial communities were not affected by elicitors. Accordingly, other authors reported no or only partial changes in leaf surface microbial composition of *Vitis vinifera* “Pinot gris” and “Pinot noir” plants following treatments with a biocontrol agent (*Lysobacter capsici*), chemical fungicide, laminarin, and a protein-based resistant inducer against downy mildew [43,44]. Likewise, only minor modifications were observed in the phyllosphere of strawberry and *Brassica oleracea* after biocontrol agent application in working field conditions [81,82].

According to PERMANOVA analysis, the structure of fungal communities was similar among all treatments. The only significant difference was observed for the *Erysiphe* genus, whose abundance was much higher in “Nebbiolo” than “Moscato” samples. Although the richness of the fungal communities of grape leaves can be affected by the adopted farming approaches (conventional or organic) [83], poor information is available on the influence of specific compounds applied to control fungal diseases. To date, *Aureobasidium pullulans* and *Epicoccum nigrum*, both biological control agents against some grapevine pathogens, are the only two species identified in grapevine belonging to *Aureobasidium* and *Epicoccum* genera [84]. Interestingly, these genera were more abundant in “Moscato” leaves, in which a lower disease severity was observed. Although amplicon-sequencing technology did not allow species identification, it was conceivable that the reads assigned to *Aureobasidium* and *Epicoccum* genera corresponded to the biological control agents, which might play a role in controlling the powdery mildew development. Moreover, results indicated that alternative treatments were able to successfully reduce the disease development, despite a high amount of reads ascribed to *Erysiphe* was found. A possible explanation of this could be that the pathogen was still present in leaf tissues but was impaired in pathogenesis. However, the molecular mechanisms underlying such reduced virulence are not clear, and this aspect certainly requires further investigation. Furthermore, it is worth noting that powdery mildew control by laminarin reached an efficacy similar to that obtained in field trials by applying the conventional sulfur treatment on “Moscato” plants [13].

In addition to biotic and abiotic factors, the genotype is an important component in shaping the size and composition of phyllosphere communities [85]. For instance, it was reported that endophytic bacterial communities differed in several cultivars of cotton [86], sweet pepper [87], tomato [88], and potato [89]. Nevertheless, the interactions between fungal communities and different genotypes are largely unknown in grapevine. Our results, through PERMANOVA and ANOVA analyses, showed a significant genotype effect on both fungal community composition and abundance of *Erysiphe* OTUs. Conversely, the treatment variable did not affect the fungal community composition (also when genotypes were considered independently), revealing a limited effect on the natural community. These data were consistent with those obtained from CLPP analysis, suggesting that genotype is the most important factor influencing the phyllosphere microbial composition. In light of this, our study was the first to provide insights into a functional relationship(s) among grape genotypes, phyllosphere microbial populations, and their potential functions in pathogen control.

Finally, as a further level of complexity, plant viruses have long been considered only as pathogens without taking into account their pivotal roles in ecology and evolution [90]. Thanks to the advances in sequencing technologies, numerous leaps have been made in virus discovery, revealing their ubiquity and abundance in living organisms and environments [91,92,93,94,95]. Regarding plant virus biology, in spite of their wide distribution, most of them do not induce visible symptoms in the host, and some mutualistic and beneficial relationships have been recently reported [96,97]. It is well known that grapevines can host a number of viral entities, most of them recognized as pathogens [74]. Today, only preventative strategies are available against viral pathogens, and a few studies have investigated molecular and physiological mechanisms triggered during virus infection events [30]. Also, in grapevine, some examples of mutual adaptation have been provided [98,99,100,101], demonstrating that some viruses are not always detrimental to the plant. These works highlighted the importance of studying plant and virus interactions considering plant genotype, viral strains, and co-infection with other viruses and environmental conditions. In our study, the higher richness of viral entities was found in “Moscato” than in “Nebbiolo” samples, offering evidence of genotype diversity matching to that observed through the analysis of the fungal community, microbial physiological profiling, and disease development. It has been previously described that grape viruses can influence plant responses to abiotic [98,99] and biotic stress, including fungal disease [100]; thus, the existence of some relevant interactions between viruses, fungal pathogens, and microbial communities is highly conceivable. Accordingly, the lower disease severity observed in “Moscato” CTRL in comparison to “Nebbiolo” CTRL might not be exclusively linked to the genotype, but, more likely, it could be the result of a tight virus-genotype interaction. Furthermore, this hypothetical virus-effect counteracting disease development seemed to be no longer relevant when the products against powdery mildew were applied. Indeed, no differences in disease severity were found between “Nebbiolo” and “Moscato” treated with elicitors, implying that viruses or virus-genotype interaction had no effect (or a limited influence) on the antifungal activity of these compounds. The proposed interaction among viruses and fungi, or even more complex interplays involving the entire holobiont, are worthy of being further addressed in the future by setting up ad hoc experiments.

## 5. Conclusions 

In recent years, the application of elicitor compounds has been gaining wide attention in the field of plant disease control techniques, being those effective and environmentally-friendly alternatives to conventional chemical pesticides.

In conclusion, the treatments here applied could represent a promising alternative to chemical fungicides against powdery mildew, combining them in an integrated or organic pest management program. A genotype effect was also observed both in disease development and phyllosphere community composition, as well as in culturable microbial physiological profiling. Independently of the treatment, “Moscato” plants were less susceptible to disease development, bringing out peculiar (endogenous) features that led to lower *Erisyphe* spread. In agreement with sequencing results, leaf indigenous communities were not affected by the treatment imposition, positively revealing their specificity in inducing resistance against the pathogen only. This result is of particular interest for pest management programs, as it is an essential requirement that the compounds used for pest control do not alter the beneficial microbial communities interacting with the plant and its surrounding environment. 

Considering the plant as a holobiont, it is worth noting the importance of investigating the microbial communities, including the viral entities that naturally reside within the plant, in order to understand physiological and ecological features directly arising from these interactions. The knowledge provided from the present study could be used in the future for orienting the development of sustainable strategies addressed to effectively manipulate plant immunity response. Additional experiments are ongoing to explore this subject more in-depth and to dissect treatment, genotype, and microorganism effects on the molecular and biochemical events associated with plant disease control.

## Figures and Tables

**Table 1:** Active ingredients and commercial formulations applied in *Vitis vinifera* “Moscato” and “Nebbiolo” inoculated with *Erysiphe necator*.

Active Ingredient (a.i.)	Commercial Product (c.p.)	a.i. Concentration	Dose of a.i. (g/ha)	Dose of c.p. (g/100 L)
Acibenzolar-S-methyl (AcS-Mt)	BION	50%	100	20
Potassium phosphonate (K-Pho)	CENTURY SL	755 g/L	3020	600
Laminarin (Lam)	VACCIPLANT	45 g/L	90	20

a.i.: active ingredient; c.p.: commercial product.

**Table 2:** Experimental outline (timing of treatments and pathogen inoculation) applied in *Vitis vinifera* “Moscato” and “Nebbiolo” vines inoculated with *Erysiphe necator*.

Treatments	10 August 2017	17 August 2017	17 August 2017	26 August 2017	4 September 2017	5 September 2017	11 September 2017	19 September 2017	22 September 2017
**Inoculated untreated control (CTRL)**	-	-	*E. necator* (1 × 10^5^ conidia/mL)	-	-	*E. necator* (1 × 10^5^ conidia/mL)	-	-	Disease scoring and Sample collection
**Acibenzolar-S-methyl (AcS-Mt)**	Bion	Bion	*E. necator* (1 × 10^5^ conidia/mL)	Bion	Bion	*E. necator* (1 × 10^5^ conidia/mL)	Bion	Bion	
**Potassium phosphonate (K-Pho)**	Century Sl	Century Sl	*E. necator* (1 × 10^5^ conidia/mL)	Century Sl	Century Sl	*E. necator* (1 × 10^5^ conidia/mL)	Century Sl	Century Sl	
**Laminarin (Lam)**	Vacciplant	Vacciplant	*E. necator* (1 × 10^5^ conidia/mL)	Vacciplant	Vacciplant	*E. necator* (1 × 10^5^ conidia/mL)	Vacciplant	Vacciplant	

**Table 3:** Microbial diversity expressed as Simpson’ and Shannon’s indices calculated on the base of Biolog^TM^ EcoPlate analysis carried out 3 days (h72) after plate incubation. Values represent mean ± standard error. CTRL—inoculated untreated control, AcS-Mt—acibenzolar-S-methyl, K-Pho—potassium phosphonate, Lam—laminarin.

Cultivar	Index	Treatment	h72
MOSCATO	Simpson index (D)	CTRL	0.97 ± 0.03
		AcS-Mt	0.96 ± 0.04
		K-Pho	0.99 ± 0.00
		Lam	0.94 ± 0.04
	Shannon index (H’)	CTRL	1.50 ± 1.27
		AcS-Mt	1.36 ± 1.20
		K-Pho	0.92 ± 0.56
		Lam	2.16 ± 1.07
NEBBIOLO	Simpson index (D)	CTRL	0.99 ± 0.00
		AcS-Mt	0.96 ± 0.02
		K-Pho	0.83 ± 0.15
		Lam	0.95 ± 0.04
	Shannon index (H’)	CTRL	1.56 ± 0.00
		AcS-Mt	2.51 ± 0.88
		K-Pho	3.16 ± 2.44
		Lam	2.29 ± 1.5

**Table 4:** List of viruses and viroids detected by RNA-seq analysis in “Moscato” and “Nebbiolo” leaves. Three biological replicates for each treatment were used. For each virus or viroid, biological replicates are represented by a “+” symbol or a “−” symbol if detectable or undetectable, respectively. CTRL—inoculated untreated control; AcS-Mt—acibenzolar-S-methyl; K-Pho—potassium phosphonate; Lam—laminarin.

	Nebbiolo				Moscato			
Virus/Viroid Name	CTRL	AcS-Mt	K-Pho	Lam	CTRL	AcS-Mt	K-Pho	Lam
Grapevine leafroll associated virus 1	−−−	−−−	−−−	−−−	+++	+++	+++	+++
Grapevine leafroll associated virus 3	−−−	−−−	−−−	−−−	++−	+−−	+−−	−−−
Grapevine ruspestris stem pitting associated virus	+++	+++	+++	+++	+++	+++	+++	+++
Grapevine pinot gris virus	++−	+−−	++−	−−−	−−−	−−−	−−−	−−−
Grapevine virus A	−−−	−−−	−−−	−−−	+++	+++	+++	+++
Grapevine virus B	−−−	−−−	−−−	−−−	−−−	−−−	++−	−−−
Grapevine virus D	−−−	−−−	−−−	−−−	+−−	−−−	−−−	−−−
Grapevine virus F	−−−	−−−	−−−	−−−	++−	−−−	−−−	−−−
Grapevine deformation virus	−−−	−−−	−−−	−−−	++−	+−−	++−	−−−
Grapevine fanleaf virus	−−−	−−−	−−−	−−−	+++	+++	++−	−−−
Grapevine syrah virus	−−−	−−−	−−−	−−−	+−−	−−−	+−−	+−−
Grapevine rupestris vein feathering virus	−−−	−−−	−−−	−−−	+++	++−	+−−	−−−
Grapevine fleck virus	+++	+++	+++	+++	+++	+++	+++	+++
Grapevine virus T	−−−	−−−	−−−	−−−	++−	++−	+−−	−−−
Grapevine yellow speckle viroid 1	+++	+++	+++	+++	+++	+++	+++	+++
Grapevine yellow speckle viroid 2	−−−	−−−	−−−	−−−	+−−	−−−	−−−	−−−
Hop stunt viroid	+++	+++	+++	+++	++−	+++	+++	+++

## Supplementary Materials

The following are available online at https://www.mdpi.com/2076-2607/7/12/662/s1.
