# Supplementary material for: Grapevine Phyllosphere Community Analysis in Response to Elicitor Application against Powdery Mildew
Source: Microorganisms. 2019 Dec 7;7(12):662. doi: 10.3390/microorganisms7120662 (PMC6956034; doi:10.3390/microorganisms7120662)

Supplementary tables and figures

Grapevine Phyllosphere Community Analysis in Response to Elicitor Application against Powdery Mildew

Luca Nerva [1,2,*], Chiara Pagliarani [2], Massimo Pugliese [3,4], Matteo Monchiero [5], Solène Gonthier [2,6], Maria Lodovica Gullino [3,4], Giorgio Gambino [2] and Walter Chitarra [1,2,*]

1 Research Centre for Viticulture and Enology, Council for Agricultural Research and Economics (CREA-VE), Via XXVIII Aprile 26, 31015 Conegliano, Italy
2 Institute for Sustainable Plant Protection, National Research Council (IPSP-CNR), Strada delle Cacce 73, 10135 Torino, Italy; chiara.pagliarani@ipsp.cnr.it (C.P.); solene.gonthier@insa-lyon.fr (S.G.); giorgio.gambino@ipsp.cnr.it (G.G.)
3 Centre of Competence for the Innovation in the Agro-Environmental Sector (AGROINNOVA), University of Torino, Largo Paolo Braccini 2, 10095 Grugliasco (TO), Italy; massimo.pugliese@unito.it (M.P.); marialodovica.gullino@unito.it (M.L.G.)
4 Department of Agricultural, Forest and Food Sciences (DISAFA), University of Torino, Largo Paolo Braccini 2, 10095 Grugliasco (TO), Italy
5 Ant-Net S.r.l., Via Livorno 60, 10140 Torino, Italy; m.monchiero@alice.it
6 Biocomputing and Modelling Department, National Institute of Applied Sciences, INSA Lyon, 69621 Villeurbanne cedex, France
* Correspondence: luca.nerva@ipsp.cnr.it (L.N.); walter.chitarra@crea.gov.it (W.C.); Tel: +39-04-3845-6712 (W.C.); Fax: +39-04-3845-0773 (W.C.)

Supplementary Table S1. Resume of read counts and statistics for each sequenced sample in the MiSeq run

Sample	Total Bases	Read Counts	GC (%)	AT (%)	Q20 (%)	Q30 (%)
CTRL1_MO_DNA_ITS	50,897,896	169,096	65.08	34.92	84.02	73.12
CTRL2_MO_DNA_ITS	50,721,510	168,510	65.30	34.70	83.70	72.87
CTRL3_MO_DNA_ITS	47,937,260	159,260	63.79	36.21	84.43	73.83
AcS-Mt1_MO_DNA_ITS	49,229,754	163,554	67.35	32.65	83.16	72.08
AcS-Mt2_MO_DNA_ITS	52,181,360	173,360	67.88	32.12	82.69	71.34
AcS-Mt3_MO_DNA_ITS	49,565,670	164,670	66.73	33.27	83.30	72.28
K-Pho1_MO_DNA_ITS	64,607,242	214,642	68.11	31.89	83.29	72.36
K-Pho2_MO_DNA_ITS	61,665,870	204,870	67.27	32.73	83.56	72.52
K-Pho3_MO_DNA_ITS	60,049,500	199,500	67.33	32.67	83.93	73.04
Lam1_MO_DNA_ITS	65,298,940	216,940	65.61	34.39	84.27	73.50
Lam2_MO_DNA_ITS	50,214,626	166,826	67.32	32.68	83.41	72.28
Lam3_MO_DNA_ITS	50,790,740	168,740	68.46	31.54	83.52	72.67
CTRL1_NE_DNA_ITS	50,250,144	166,944	63.98	36.02	83.28	72.33
CTRL2_NE_DNA_ITS	61,148,752	203,152	63.38	36.62	84.50	73.57
CTRL3_NE_DNA_ITS	58,818,410	195,410	64.46	35.54	83.29	72.17
AcS-Mt1_NE_DNA_ITS	55,802,992	185,392	67.0	33.0	82.60	71.31
AcS-Mt2_NE_DNA_ITS	51,794,876	172,076	66.12	33.88	83.0	71.78
AcS-Mt3_NE_DNA_ITS	56,359,842	187,242	66.57	33.43	82.90	71.80
K-Pho1_NE_DNA_ITS	60,026,624	199,424	65.96	34.04	81.36	69.48
K-Pho2_NE_DNA_ITS	63,471,870	210,870	64.90	35.10	82.88	71.92
K-Pho3_NE_DNA_ITS	53,839,268	178,868	67.06	32.94	82.61	71.44
Lam1_NE_DNA_ITS	57,239,966	190,166	65.36	34.64	84.31	73.44
Lam2_NE_DNA_ITS	54,599,594	181,394	64.01	35.99	83.99	73.06
Lam3_NE_DNA_ITS	51,449,328	170,928	65.75	34.25	82.74	71.41

Supplementary Table S2. Abundances of TOP 10 fungal genera among the analyzed samples

	NE CTRL			NE AcS-Mt			NE K-Pho			NE Lam			MO CTRL			MO AcS-Mt			MO K-Pho			MO Lam		
**Genera**	**Mean**		**SD**	**Mean**		**SD**	**Mean**		**SD**	**Mean**		**SD**	**Mean**		**SD**	**Mean**		**SD**	**Mean**		**SD**	**Mean**		**SD**
Erysiphe	77.71%	±	6.86%	74.73%	±	5.31%	67.44%	±	10.29%	86.71%	±	2.11%	50.78%	±	10.45%	22.28%	±	6.18%	21.52%	±	6.61%	38.90%	±	5.97%
Alternaria	10.08%	±	2.88%	10.39%	±	4.43%	13.48%	±	3.28%	1.02%	±	0.55%	20.72%	±	2.81%	35.33%	±	3.58%	32.87%	±	3.88%	22.52%	±	1.90%
Cladosporium	5.23%	±	0.83%	5.10%	±	0.04%	7.09%	±	2.58%	3.75%	±	1.14%	10.31%	±	4.63%	12.40%	±	3.72%	12.98%	±	2.06%	16.43%	±	2.13%
Epicoccum	5.27%	±	1.97%	6.55%	±	0.66%	8.56%	±	1.14%	2.48%	±	1.33%	12.19%	±	1.21%	13.28%	±	3.09%	24.58%	±	3.90%	10.48%	±	1.13%
Aureobasidium	0.26%	±	0.10%	0.22%	±	0.13%	0.72%	±	0.20%	0.16%	±	0.06%	0.92%	±	0.51%	10.01%	±	1.79%	2.25%	±	1.10%	2.16%	±	0.74%
Bipolaris	0.56%	±	0.29%	0.53%	±	0.44%	0.43%	±	0.19%	0.46%	±	0.30%	1.69%	±	0.55%	2.32%	±	0.43%	1.86%	±	0.26%	5.44%	±	0.29%
Pithomyces	0.32%	±	0.11%	1.52%	±	0.26%	1.29%	±	0.28%	0.85%	±	0.40%	1.86%	±	0.76%	2.44%	±	1.44%	1.91%	±	0.46%	0.96%	±	0.38%
Filobasidium	0.12%	±	0.07%	0.02%	±	0.01%	0.20%	±	0.04%	0.03%	±	0.01%	0.89%	±	0.52%	0.25%	±	0.13%	0.20%	±	0.08%	0.07%	±	0.03%
Curvularia	0.22%	±	0.12%	0.26%	±	0.17%	0.28%	±	0.06%	0.11%	±	0.09%	0.15%	±	0.10%	0.50%	±	0.06%	0.27%	±	0.11%	0.91%	±	0.10%
Nigrospora	0.22%	±	0.19%	0.68%	±	0.07%	0.50%	±	0.29%	0.43%	±	0.16%	0.49%	±	0.11%	1.20%	±	0.36%	1.55%	±	0.96%	2.12%	±	0.74%

Supplementary Table S3. List of OTUs identified in 'Moscato' (MO) samples. Values are the mean of three biological replicates ± standard deviation (SD). MO CTRL = non-treated inoculated plants; MO AcS-Mt = plants treated with Acibenzolar-S-methyl (Bion, Syngenta Crop Protection); MO K-Pho = plants treated with Potassium phosphonate (Century, BASF Agro); MO Lam = inoculated plants treated with Laminarin (Vacciplant, Arysta Lifescience).

	MO CTRL			MO AcS-Mt			MO K-Pho			MO Lam		
	Mean		SD	Mean		SD	Mean		SD	Mean		SD
Erysiphe	49.96%	±	30.29%	21.84%	±	17.63%	19.84%	±	17.82%	38.47%	±	27.95%
Epicoccum	11.87%	±	9.83%	13.09%	±	3.19%	22.37%	±	3.78%	10.34%	±	7.16%
Alternaria	20.26%	±	12.48%	34.75%	±	13.19%	29.84%	±	12.84%	22.08%	±	7.27%
Cladosporium	10.08%	±	4.45%	12.18%	±	3.52%	11.76%	±	1.39%	16.05%	±	11.58%
Aureobasidium	0.90%	±	0.50%	9.99%	±	15.78%	2.06%	±	1.07%	2.16%	±	3.74%
Nigrospora	0.48%	±	0.41%	1.18%	±	0.36%	1.41%	±	0.90%	2.07%	±	1.95%
Claviceps	0.00%	±	0.00%	0.00%	±	0.00%	0.57%	±	0.95%	0.00%	±	0.00%
Phoma	0.00%	±	0.00%	0.00%	±	0.00%	0.62%	±	0.90%	0.00%	±	0.00%
Bipolaris	1.65%	±	1.50%	2.28%	±	0.45%	1.70%	±	0.24%	5.42%	±	8.29%
Coniozyma	0.00%	±	0.00%	0.00%	±	0.00%	0.32%	±	0.56%	0.00%	±	0.00%
Hannaella	0.12%	±	0.22%	0.14%	±	0.24%	0.55%	±	0.34%	0.00%	±	0.00%
Curvularia	0.15%	±	0.20%	0.49%	±	0.66%	0.24%	±	0.26%	0.89%	±	0.79%
Ulocladium	0.00%	±	0.00%	0.00%	±	0.00%	0.23%	±	0.23%	0.90%	±	1.51%
Pithomyces	1.82%	±	0.73%	2.39%	±	1.40%	1.69%	±	1.22%	0.93%	±	1.33%
Fusarium	0.00%	±	0.00%	0.41%	±	0.49%	0.39%	±	0.53%	0.06%	±	0.05%
Lophiostoma	0.00%	±	0.00%	0.00%	±	0.00%	0.24%	±	0.37%	0.00%	±	0.00%
Blumeria	0.00%	±	0.00%	0.00%	±	0.00%	0.00%	±	0.00%	0.12%	±	0.10%
Candida	0.00%	±	0.00%	0.12%	±	0.20%	0.00%	±	0.00%	0.00%	±	0.00%
Filobasidium	0.87%	±	1.01%	0.24%	±	0.42%	0.17%	±	0.23%	0.00%	±	0.00%
Leptosphaerulina	0.44%	±	0.75%	0.00%	±	0.00%	0.00%	±	0.00%	0.00%	±	0.00%
Microsphaeropsis	0.00%	±	0.00%	0.30%	±	0.37%	0.76%	±	0.82%	0.00%	±	0.00%
Noosia	0.00%	±	0.00%	0.00%	±	0.00%	0.14%	±	0.12%	0.00%	±	0.00%
Periconia	0.34%	±	0.47%	0.12%	±	0.21%	0.00%	±	0.00%	0.00%	±	0.00%
Pleochaeta	0.30%	±	0.07%	0.00%	±	0.00%	0.00%	±	0.00%	0.00%	±	0.00%
Podospora	0.00%	±	0.00%	0.00%	±	0.00%	0.00%	±	0.00%	0.45%	±	0.54%
Preussia	0.42%	±	0.44%	0.34%	±	0.42%	0.22%	±	0.39%	0.00%	±	0.00%
Stemphylium	0.15%	±	0.13%	0.00%	±	0.00%	4.51%	±	7.80%	0.00%	±	0.00%
Symmetrospora	0.17%	±	0.26%	0.00%	±	0.00%	0.00%	±	0.00%	0.00%	±	0.00%
Trichoderma	0.00%	±	0.00%	0.15%	±	0.11%	0.00%	±	0.00%	0.05%	±	0.09%
Vishniacozyma	0.00%	±	0.00%	0.00%	±	0.00%	0.36%	±	0.44%	0.00%	±	0.00%

Supplementary Table S4. List of OTUs identified in 'Nebbiolo' (NE) samples. Values are the mean of three biological replicates ± standard deviation (SD). CTRL = non-treated inoculated plants; AcS-Mt = plants treated with Acibenzolar-S-methyl (Bion, Syngenta Crop Protection); K-Pho = plants treated with Potassium phosphonate (Century, BASF Agro); Lam = inoculated plants treated with Laminarin (Vacciplant, Arysta Lifescience).

	NE CTRL			NE AcS-Mt			NE K-Pho			NE Lam		
	Mean		SD	Mean		SD	Mean		SD	Mean		SD
Erysiphe	68.76%	±	24.88%	30.32%	±	1.98%	36.37%	±	20.44%	63.68%	±	18.73%
Alternaria	7.76%	±	1.84%	4.39%	±	2.28%	5.92%	±	1.97%	3.60%	±	0.57%
Cladosporium	4.58%	±	1.73%	2.08%	±	0.26%	3.44%	±	1.52%	2.67%	±	0.80%
Epicoccum	4.30%	±	0.99%	2.68%	±	0.53%	4.26%	±	1.42%	1.69%	±	0.56%
Aureobasidium	0.23%	±	0.17%	0.09%	±	0.06%	0.48%	±	0.69%	0.11%	±	0.04%
Bipolaris	0.51%	±	0.37%	0.23%	±	0.21%	0.22%	±	0.23%	0.38%	±	0.31%
Pithomyces	0.31%	±	0.25%	0.63%	±	0.17%	0.69%	±	0.34%	0.58%	±	0.15%
Filobasidium	0.12%	±	0.17%	0.01%	±	0.01%	0.14%	±	0.17%	0.02%	±	0.01%
Curvularia	0.22%	±	0.24%	0.10%	±	0.05%	0.09%	±	0.10%	0.08%	±	0.08%
Nigrospora	0.21%	±	0.21%	0.28%	±	0.03%	0.19%	±	0.16%	0.31%	±	0.11%
Pleochaeta	0.15%	±	0.17%	0.05%	±	0.05%	0.13%	±	0.12%	0.13%	±	0.10%
Ulocladium	0.14%	±	0.06%	0.06%	±	0.06%	0.01%	±	0.02%	0.09%	±	0.11%
Blumeria	0.08%	±	0.01%	0.03%	±	0.01%	0.04%	±	0.03%	0.08%	±	0.05%
Hannaella	0.11%	±	0.07%	0.00%	±	0.01%	0.04%	±	0.04%	0.03%	±	0.03%
Leptosphaerulina	0.04%	±	0.03%	0.03%	±	0.03%	0.02%	±	0.01%	0.04%	±	0.05%
Vishniacozyma	0.02%	±	0.03%	0.00%	±	0.01%	0.18%	±	0.29%	0.01%	±	0.02%
Fusarium	0.06%	±	0.06%	0.07%	±	0.05%	0.44%	±	0.64%	0.14%	±	0.12%
Periconia	0.02%	±	0.02%	0.09%	±	0.10%	0.15%	±	0.20%	0.03%	±	0.02%
Microsphaeropsis	0.04%	±	0.04%	0.05%	±	0.05%	0.19%	±	0.18%	0.00%	±	0.00%
Stemphylium	0.03%	±	0.03%	0.01%	±	0.03%	0.02%	±	0.02%	0.05%	±	0.05%
Sordaria	0.00%	±	0.00%	0.10%	±	0.17%	0.00%	±	0.00%	0.00%	±	0.00%

Supplementary Table S5. Bray-Curtis matrix was used to perform PERMANOVA analysis to highlight the genotype effect. All samples from each genotype were considered independently of treatment type. *p* (Bonferroni-corrected) and *F* values for comparison of 'Nebbiolo' (NE) and 'Moscato' (MO) samples are reported below the table.

	NE	MO
P value		
NE		0.0001
MO	0.0001	

F: 23.16

Supplementary Table S6. Bray-Curtis matrix was used to perform PERMANOVA analysis to assess possible effects of treatment on 'Nebbiolo' (NE). *p* (Bonferroni-corrected) and *F* values are reported below the table. CTRL = non-treated inoculated plants; AcS-Mt = plants treated with Acibenzolar-S-methyl (Bion, Syngenta Crop Protection); K-Pho = plants treated with Potassium phosphonate (Century, BASF Agro); Lam = inoculated plants treated with Laminarin (Vacciplant, Arysta Lifescience).

	NE CTRL	NE AcS-Mt	NE K-Pho	NE Lam
P value				
NE CTRL		0.499	0.2074	0.1042
NE AcS-Mt	0.499		0.3988	0.1055
NE K-Pho	0.2074	0.3988		0.101
NE Lam	0.1042	0.1055	0.101	
F value				
NE CTRL		0.5514	1.526	3.382
NE Ac-Mt	0.5514		0.8985	9.57
NE K-Pho	1.526	0.8985		7.114
NE Lam	3.382	9.57	7.114	

Supplementary Table S7. Bray-Curtis matrix was used to perform PERMANOVA analysis to assess possible effects of treatment on 'Moscato' (MO). *p* (Bonferroni-corrected) and *F* values are reported below the table. CTRL = non-treated inoculated plants; AcS-Mt = plants treated with Acibenzolar-S-methyl (Bion, Syngenta Crop Protection); K-Pho = plants treated with Potassium phosphonate (Century, BASF Agro); Lam = inoculated plants treated with Laminarin (Vacciplant, Arysta Lifescience).

	MO CTRL	MO AcS-Mt	MO K-Pho	MO Lam
P value				
MO CTRL		0.2999	0.3025	0.6088
MO AcS-Mt	0.2999		0.7008	0.6097
MO K-Pho	0.3025	0.7008		0.4067
MO Lam	0.6088	0.6097	0.4067	
F value				
MO CTRL		1.67	1.874	0.2504
MO Ac-Mt	1.67		0.4393	0.8533
MO K-Pho	1.874	0.4393		1.055
MO Lam	0.2504	0.8533	1.055	

Supplementary Table S8. Bray-Curtis matrix was used to perform PERMANOVA analysis in order to compare treatment effects on 'Nebbiolo' (NE) and 'Moscato' (MO) samples at once. *p* (Bonferroni-corrected) and *F* values are reported below the table. CTRL = non-treated inoculated plants; AcS-Mt = plants treated with Acibenzolar-S-methyl (Bion, Syngenta Crop Protection); K-Pho = plants treated with Potassium phosphonate (Century, BASF Agro); Lam = inoculated plants treated with Laminarin (Vacciplant, Arysta Lifescience).

	NE CTRL	NE AcS-Mt	NE K-Pho	NE Lam	MO CTRL	MO AcS-Mt	MO K-Pho	MO Lam
P value								
NE CTRL		0.0988	0.1941	0.7985	0.0946	0.1058	0.1019	0.2002
NE AcS-Mt	0.0988		0.7074	0.1035	0.405	0.0978	0.0989	0.4068
NE K-Pho	0.1941	0.7074		0.1998	0.3083	0.102	0.0988	0.2991
NE Lam	0.7985	0.1035	0.1998		0.1002	0.0999	0.0984	0.1967
MO CTRL	0.0946	0.405	0.3083	0.1002		0.3067	0.2997	1
MO AcS-Mt	0.1058	0.0978	0.102	0.0999	0.3067		0.7905	0.9029
MO K-Pho	0.1019	0.0989	0.0988	0.0984	0.2997	0.7905		0.7969
MO Lam	0.2002	0.4068	0.2991	0.1967	1	0.9029	0.7969	
F value								
NE CTRL		8.552	1.672	0.8406	3.89	10.54	10.45	3.399
NE Ac-Mt	8.552		0.6059	10.94	1.981	9.368	10.54	2.335
NE K-Pho	1.672	0.6059		2.146	1.083	4.463	4.413	1.72
NE Lam	0.8406	10.94	2.146		4.373	13.6	15.03	3.408
MO CTRL	3.89	1.981	1.083	4.373		1.054	1.215	0.3847
MO Ac-Mt	10.54	9.368	4.463	13.6	1.054		0.4001	0.5917
MO K-Pho	10.45	10.54	4.413	15.03	1.215	0.4001		0.7494
MO Lam	3.399	2.335	1.72	3.408	0.3847	0.5917	0.7494	

Supplementary Table S9. Alpha diversity indices calculated starting from ITS sequencing data obtained for each treatment in 'Nebbiolo' (NE) and 'Moscato' (MO). CTRL = non-treated inoculated plants; AcS-Mt = plants treated with Acibenzolar-S-methyl (Bion, Syngenta Crop Protection); K-Pho = plants treated with Potassium phosphonate (Century, BASF Agro); Lam = inoculated plants treated with Laminarin (Vacciplant, Arysta Lifescience).

	NE CTRL			NE AcS-Mt			NE K-Pho			NE Lam			MO CTRL			MO AcS-Mt			MO K-Pho			MO Lam		
	Mean		SD	Mean		SD	Mean		SD	Mean		SD	Mean		SD	Mean		SD	Mean		SD	Mean		SD
**Simpson (D)**	0.38	±	0.09	0.44	±	0.06	0.53	±	0.11	0.26	±	0.04	0.60	±	0.28	0.74	±	0.05	0.76	±	0.05	0.69	±	0.19
**Shannon (H')**	0.87	±	0.13	1.01	±	0.10	1.18	±	0.16	0.65	±	0.09	1.37	±	0.60	1.64	±	0.10	1.72	±	0.17	1.49	±	0.45
**Chao**	20.33	±	1.53	20.33	±	1.00	20.67	±	3.21	19.67	±	1.00	20.33	±	3.61	19.67	±	2.31	19.67	±	2.08	20.00	±	2.00

Supplementary Table S10. Resume of read counts and statistics for each sequenced sample in the NovaSeq run.

Sample	Read Counts	GC (%)	AT (%)	Q20 (%)	Q30 (%)
1-1_MO_RNA	51,442,584	46.75	53.25	98.74	95.99
1-1_NE_RNA	49,070,822	46.68	53.32	98.75	95.97
1-2_MO_RNA	45,950,076	46.20	53.80	98.68	95.78
1-2_NE_RNA	52,359,574	46.09	53.91	98.73	95.96
1-3_MO_RNA	44,637,026	46.27	53.74	98.72	95.93
1-3_NE_RNA	105,207,854	46.46	53.54	98.62	95.62
2-1_MO_RNA	43,016,122	46.40	53.60	98.75	96.0
2-1_NE_RNA	99,822,934	45.94	54.06	98.70	95.82
2-2_MO_RNA	45,539,430	48.83	51.17	98.71	95.88
2-2_NE_RNA	98,299,580	45.87	54.13	98.76	95.93
2-3_MO_RNA	50,716,752	46.26	53.74	98.66	95.85
2-3_NE_RNA	98,607,242	45.75	54.26	98.69	95.75
3-1_MO_RNA	49,042,662	46.46	53.54	98.67	95.82
3-1_NE_RNA	82,768,782	45.87	54.13	98.59	95.61
3-2_MO_RNA	51,651,078	46.35	53.65	98.85	96.31
3-2_NE_RNA	87,883,904	46.60	53.40	98.59	95.59
3-3_MO_RNA	54,955,000	46.32	53.68	98.84	96.34
3-3_NE_RNA	52,636,722	47.58	52.42	98.66	95.84
4-1_MO_RNA	46,153,778	46.05	53.95	98.86	96.30
4-1_NE_RNA	53,996,182	46.73	53.27	98.67	95.93
4-2_MO_RNA	42,770,800	46.44	53.57	98.83	96.33
4-2_NE_RNA	53,019,634	46.91	53.09	98.69	95.81
4-3_MO_RNA	109,690,716	46.39	53.61	98.85	96.19
4-3_NE_RNA	47,729,898	46.34	53.66	98.71	95.92
5-1_MO_RNA	94,239,798	46.40	53.60	98.68	95.97
5-1_NE_RNA	48,834,504	46.25	53.75	98.78	96.07
5-2_MO_RNA	99,231,268	46.44	53.56	98.74	96.03
5-2_NE_RNA	41,094,770	45.74	54.26	98.75	95.92
5-3_MO_RNA	88,308,474	46.46	53.55	98.68	95.94

5-3_NE_RNA	55,769,788	46.39	53.61	98.70	95.89

Supplementary Table S11. List of accession numbers retrieved from NCBI following integration with the custom database (see Methods section) for the detection of viruses, viroids and phytoplasmas.

Accession	Virus / Viroid / Phitoplasma name				
NC_022002.1	Grapevine red blotch-associated virus	NC_002692.1	Tomato mosaic virus	NC_011535.1	Grapevine Algerian latent virus
NC_015784.2	Grapevine vein-clearing virus	LT608395.1	Artichoke italian latent virus RNA 1	AY500881.1	Petunia asteroid mosaic virus
NC_027131.1	Grapevine roditis leaf discoloration-associated virus	LT608396.1	Artichoke italian latent virus RNA 2	NC_031692.1	Grapevine asteroid mosaic-associated virus
NC_035939.1	Grapevine Cabernet Sauvignon reovirus NSS	NC_005289.1	Broad bean wilt virus 1 RNA 1	NC_012484.1	Grapevine Syrah Virus-1
NC_035938.1	Grapevine Cabernet Sauvignon reovirus P7	NC_005290.1	Broad bean wilt virus 1 RNA 2	KM491303.1	Grapevine Red Globe virus
NC_035937.1	Grapevine Cabernet Sauvignon reovirus P6	NC_006057.1	Arabis mosaic virus RNA 1	NC_034205.1	Grapevine rupestris vein feathering virus
NC_035936.1	Grapevine Cabernet Sauvignon reovirus P4	NC_006056.1	Arabis mosaic virus RNA 2	NC_003347.1	Grapevine fleck virus
NC_035935.1	Grapevine Cabernet Sauvignon reovirus P1	NC_015414.1	Cherry leaf roll virus RNA 1	FJ915122.1	Blackberry virus S
NC_027802.1	Grapevine Cabernet Sauvignon reovirus segment 9	NC_015415.1	Cherry leaf roll virus RNA 2	NC_003739.1	Raspberry bushy dwarf virus RNA 1
NC_027816.1	Grapevine Cabernet Sauvignon reovirus P2	U20622.1	Blueberry leaf mottle virus RNA1	NC_003740.1	Raspberry bushy dwarf virus RNA 2
NC_027810.1	Grapevine Cabernet Sauvignon reovirus P10a	U20621.1	Blueberry leaf mottle virus RNA2	NC_011187.1	Rubus chlorotic mottle virus
NC_027809.1	Grapevine Cabernet Sauvignon reovirus P5	NC_015492.1	Grapevine Bulgarian latent virus RNA 1	KX949574.1	Southern tomato virus
NC_027808.1	Grapevine Cabernet Sauvignon reovirus P3a	NC_015493.1	Grapevine Bulgarian latent virus RNA 2	KX962563.1	Grapevine fabavirus RNA 1
NC_019493.1	Grapevine endophyte endornavirus	NC_018383.1	Grapevine Anatolian ringspot virus RNA 1	KX962564.1	Grapevine fabavirus RNA 2
NC_011705.1	Raphanus sativus cryptic virus 3 segment 1	NC_018384.1	Grapevine Anatolian ringspot virus RNA 2	HG939487.1	Tomato black ring virus RNA 1
NC_011706.1	Raphanus sativus cryptic virus 3 segment 2	NC_017939.1	Grapevine deformation virus RNA1	KX977561.1	Tomato black ring virus RNA 2
S63913.1	Beet cryptic virus 3	NC_017938.1	Grapevine deformation virus RNA2	KX645875.2	Grapevine enamovirus-1
NC_002050.1	Tomato spotted wilt virus RNA M	NC_003622.1	Grapevine chrome mosaic virus RNA 1	GQ845002.2	Sowbane mosaic virus
NC_002051.1	Tomato spotted wilt virus RNA S	NC_003621.1	Grapevine chrome mosaic virus RNA 2	JX658571.1	Grapevine partitivirus
NC_002052.1	Tomato spotted wilt virus RNA L	NC_003615.1	Grapevine fanleaf virus RNA 1	NC_035203.1	Grapevine virus T
NC_029783.1	Grapevine leafroll-associated virus 13	NC_003623.1	Grapevine fanleaf virus RNA 2	MF781081.1	Grapevine virus G
NC_007448.1	Grapevine leafroll-associated virus 2	NC_034214.1	Peach rosette mosaic virus RNA 1	MF521889.1	Grapevine virus H
NC_004667.1	Grapevine leafroll-associated virus 3	NC_034215.1	Peach rosette mosaic virus RNA 2	NC_037058.1	Grapevine virus I
NC_016509.1	Grapevine leafroll-associated virus 1	NC_005266.1	Raspberry ringspot virus RNA 1	MG637048.1	Grapevine virus J
NC_016436.1	Grapevine leafroll-associated virus 7	NC_005267.1	Raspberry ringspot virus RNA 2	NC_035480.1	Wild vitis virus 1
NC_016081.1	Grapevine leafroll-associated virus 5	NC_005097.1	Tobacco ringspot virus RNA 1	KX950822.1	Grapevine geminivirus A
NC_011702.1	Grapevine leafroll-associated virus 10	NC_005096.1	Tobacco ringspot virus RNA 2	KJ955447.1	Temperate fruit decay-associated virus
NC_016417.1	Grapevine leafroll-associated virus 6	NC_003840.1	Tomato ringspot virus RNA 1	NC_015784.2	Grapevine vein-clearing virus
NC_016416.1	Grapevine leafroll-associated virus 4	NC_003839.2	Tomato ringspot virus RNA 2	MF781082.1	Grapevine badnavirus 1
NC_011620.1	Potato virus X	NC_006964.1	Strawberry latent ringspot virus RNA 1	NC_001920.1	Grapevine yellow speckle viroid 1
NC_001948.1	Rupestris stem pitting associated virus-1	NC_006965.1	Strawberry latent ringspot virus RNA 2	NC_003612.1	Grapevine yellow speckle viroid 2
NC_015220.1	Grapevine berry inner necrosis virus	NC_001495.1	Alfalfa mosaic virus RNA 1	NC_003553.1	Australian grapevine viroid
NC_015782.1	Grapevine Pinot gris virus	NC_002024.2	Alfalfa mosaic virus RNA 2	NC_001351.1	Hop stunt viroid

NC_003604.2	Grapevine virus A	NC_002025.1	Alfalfa mosaic virus RNA 3	NC_001464.1	Citrus exocortis viroid
NC_003602.1	Grapevine virus B	NC_002034.1	Cucumber mosaic virus RNA 1	JQ046414.1	Grapevine yellows phytoplasma 16S
MF774336.1	Grapevine virus D	NC_002035.1	Cucumber mosaic virus RNA 2	JQ900580.1	Grapevine yellows phytoplasma rpl22
NC_011106.1	Grapevine virus E	NC_001440.1	Cucumber mosaic virus RNA 3	AF385627.1	Flavescence doree phytoplasma rpl22
NC_018458.1	Grapevine virus F	AY590305.1	Grapevine angular mosaic virus	AF396951.1	Flavescence doree phytoplasma rps3
NC_003397.1	Bean common mosaic virus	NC_001265.2	Carnation mottle virus	JQ181540.1	Bois noir' phytoplasma 16S
NC_001367.1	Tobacco mosaic virus	NC_003487.1	Tobacco necrosis virus D		

Figure S1. PCA analysis of EcoPlate™ data. Distinctions between cultivar 'Moscato' (MO) and 'Nebbiolo' (NE) as revealed by PCA over the time of plate incubation from day 1 (h24) to 5 (h120). Numbers reported after the name of the cultivar (NE: 'Nebbiolo', MO: 'Moscato') refer to the treatment-type as following stated: 1 = Inoculated untreated control (CTRL), 2 = Acibenzolar-S-methyl (AcS-Mt), 3 = Potassium Phosphonate (K-Pho), and 4 = Laminarin (Lam).

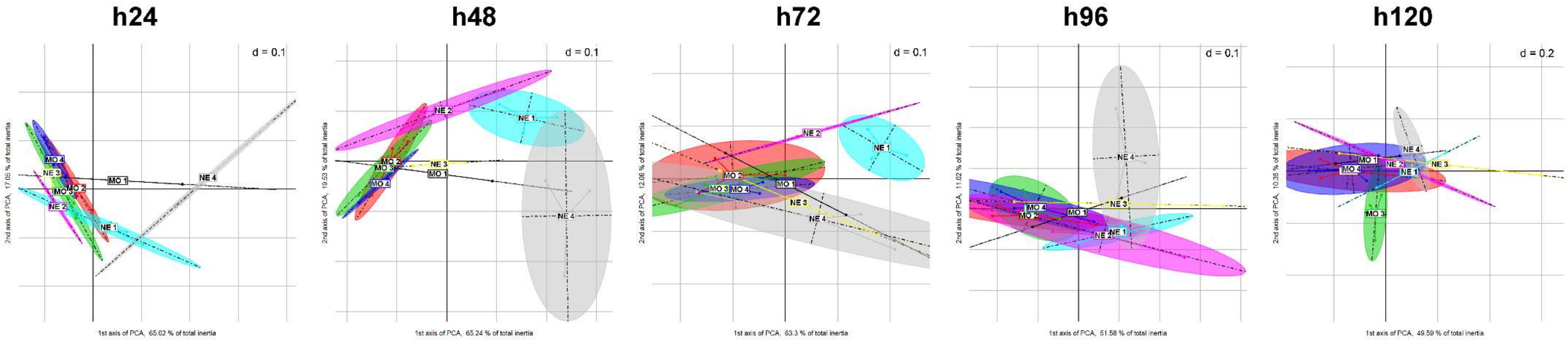

Figure S2. PCA analysis of EcoPlate™ data. Distinctions between cultivar 'Moscato' (MO) and 'Nebbiolo' (NE) compared within each treatment as revealed by PCA over the time of plate incubation from day 1 (h24) to 5 (h120). (**a**) Inoculated untreated control (CTRL), (**b**) Acibenzolar-S-methyl (AcS-Mt), (**c**) Potassium Phosphonate (K-Pho), and (**d**) Laminarin (Lam).

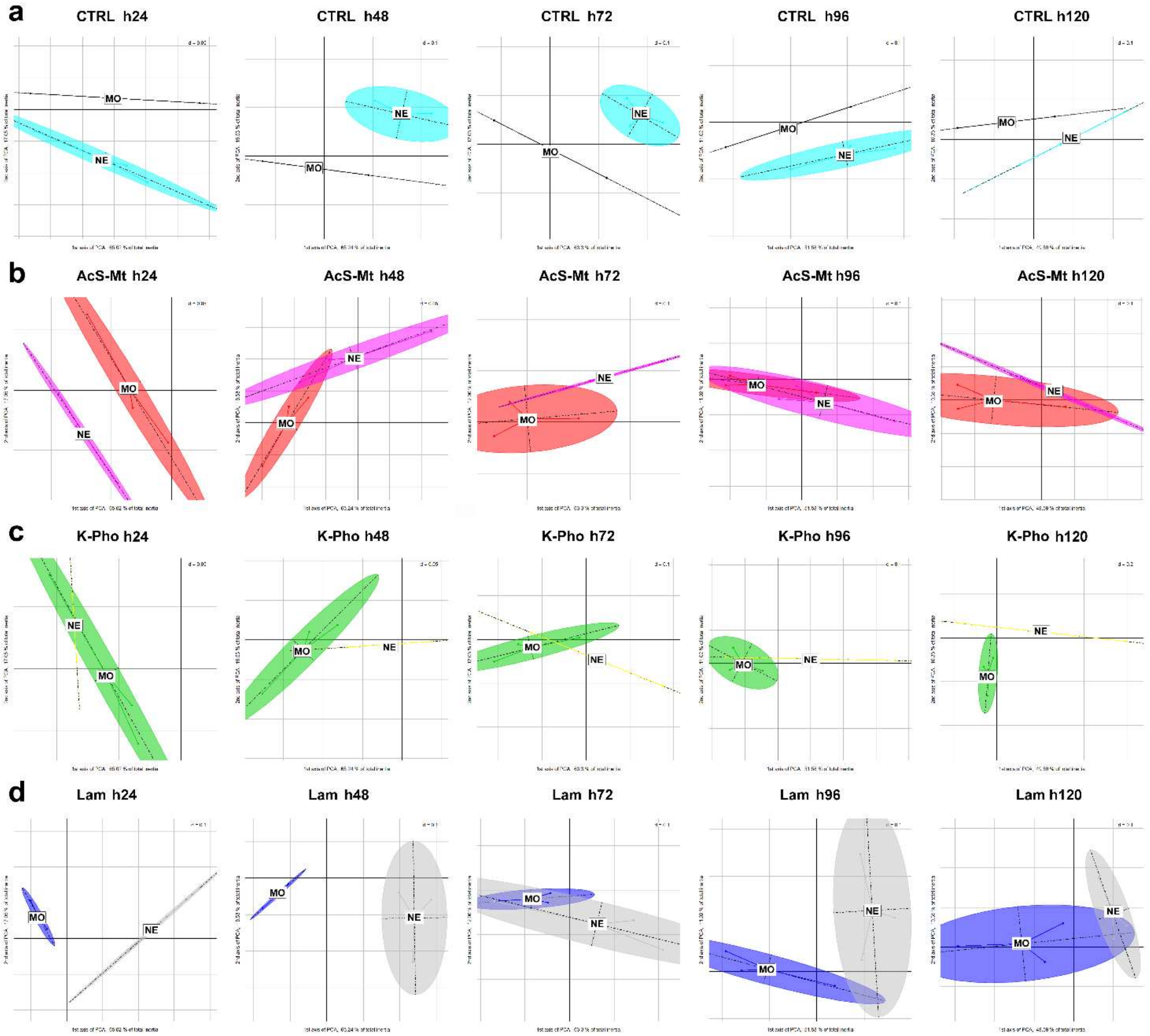

Figure S3. Average Well Colour Development (AWCD) in Biolog EcoPlates™ inoculated with 'Moscato' (a) and 'Nebbiolo' (b) leaf sample extracts. Values are calculated on the base of absorbance readings taken at 590 nm for each plate over a time course of 5 days (h24 to h120). Data represent mean ± standard deviation of three biological replicates. Inoculated untreated control (CTRL), Acibenzolar-S-methyl (AcS-Mt), Potassium Phosphonate (K-Pho), and Laminarin (Lam).

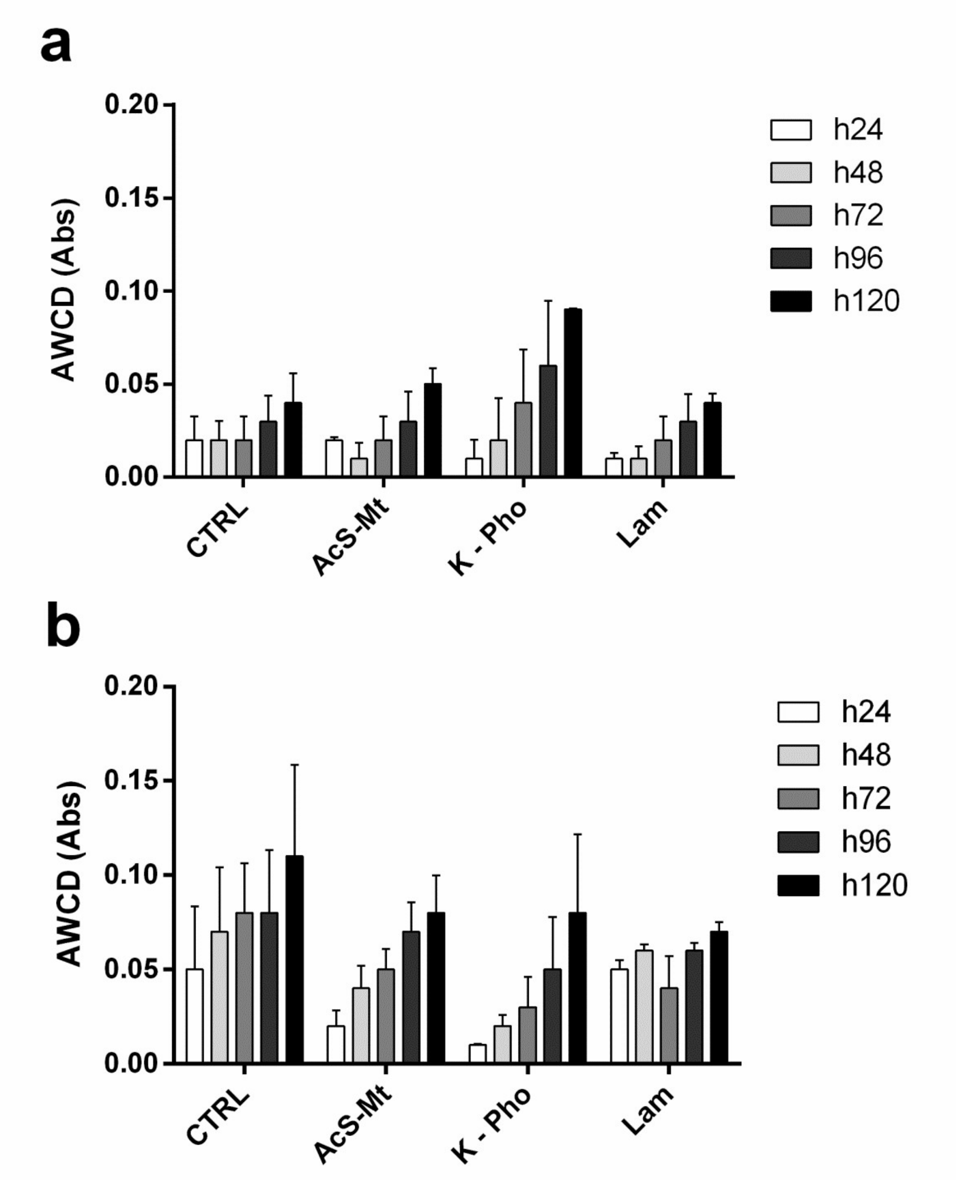

Figure S4. Rarefaction OTU curves obtained for each biological replicate of 'Moscato' (MO, panels from a to d) and 'Nebbiolo' (NE, panels from e to h) leaf samples collected from untreated (CTRL) and treated (AcS-Mt = acibenzolar-S-methyl, K-Pho = Potassium Phosphonate, Lam = Laminarin) plants infected by powdery mildew.

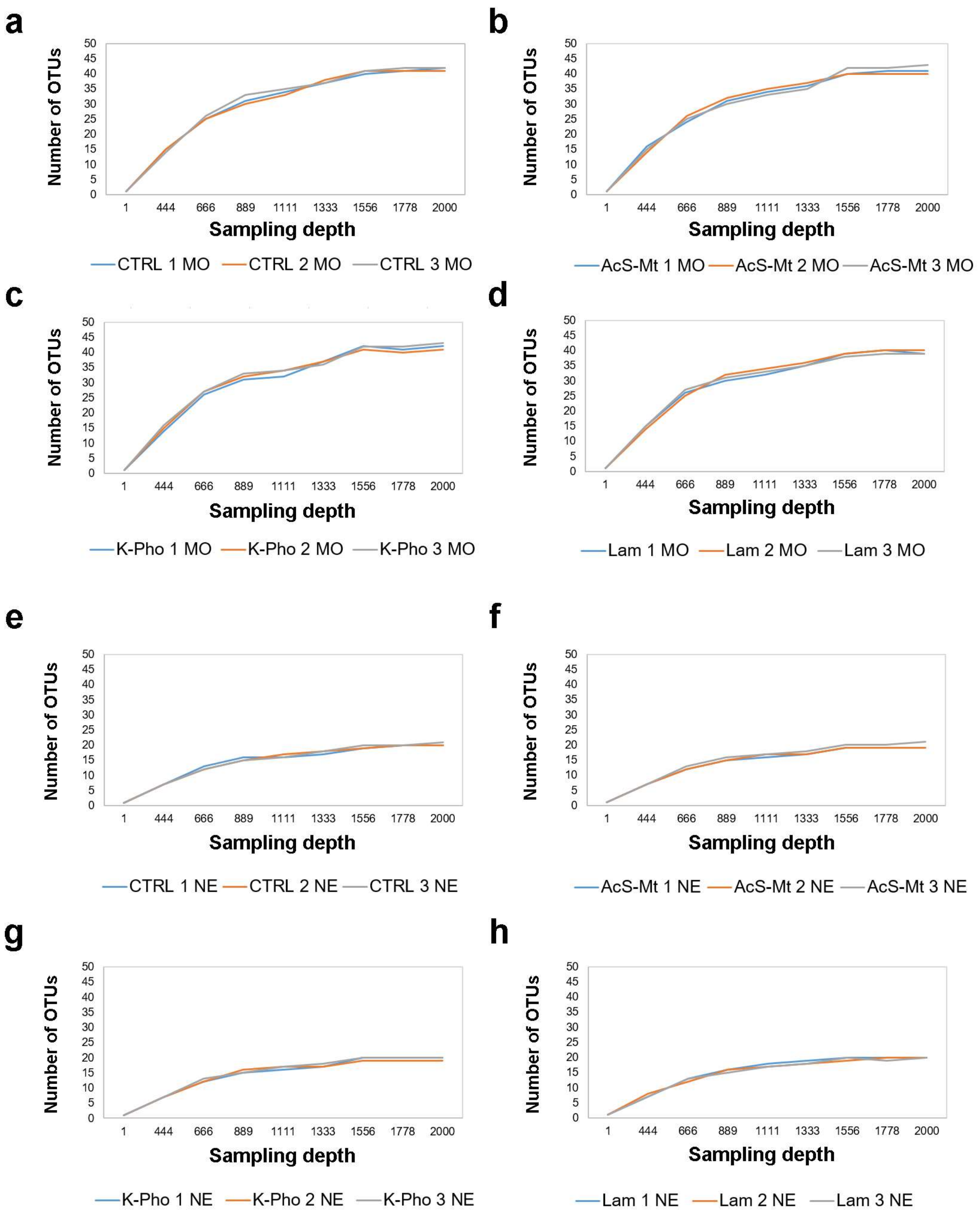

Figure S5. Agarose gel electrophoretic analysis of DNA fragments amplified by multiplex RT-PCR for detection of virus (**a** and **b**, Gambino 2015), viroids (**b**, Hajizadeh et al., 2012) and Grapevine pinot gris virus, GPGV (**c**, Glasa et al., 2014) in 'Moscato' and 'Nebbiolo'. CTRL - Inoculated untreated control, AcS-Mt - Acibenzolar-S-methyl, K-Pho - Potassium Phosphonate, LAM – Laminarin. NE: 'Nebbiolo', MO: 'Moscato'.

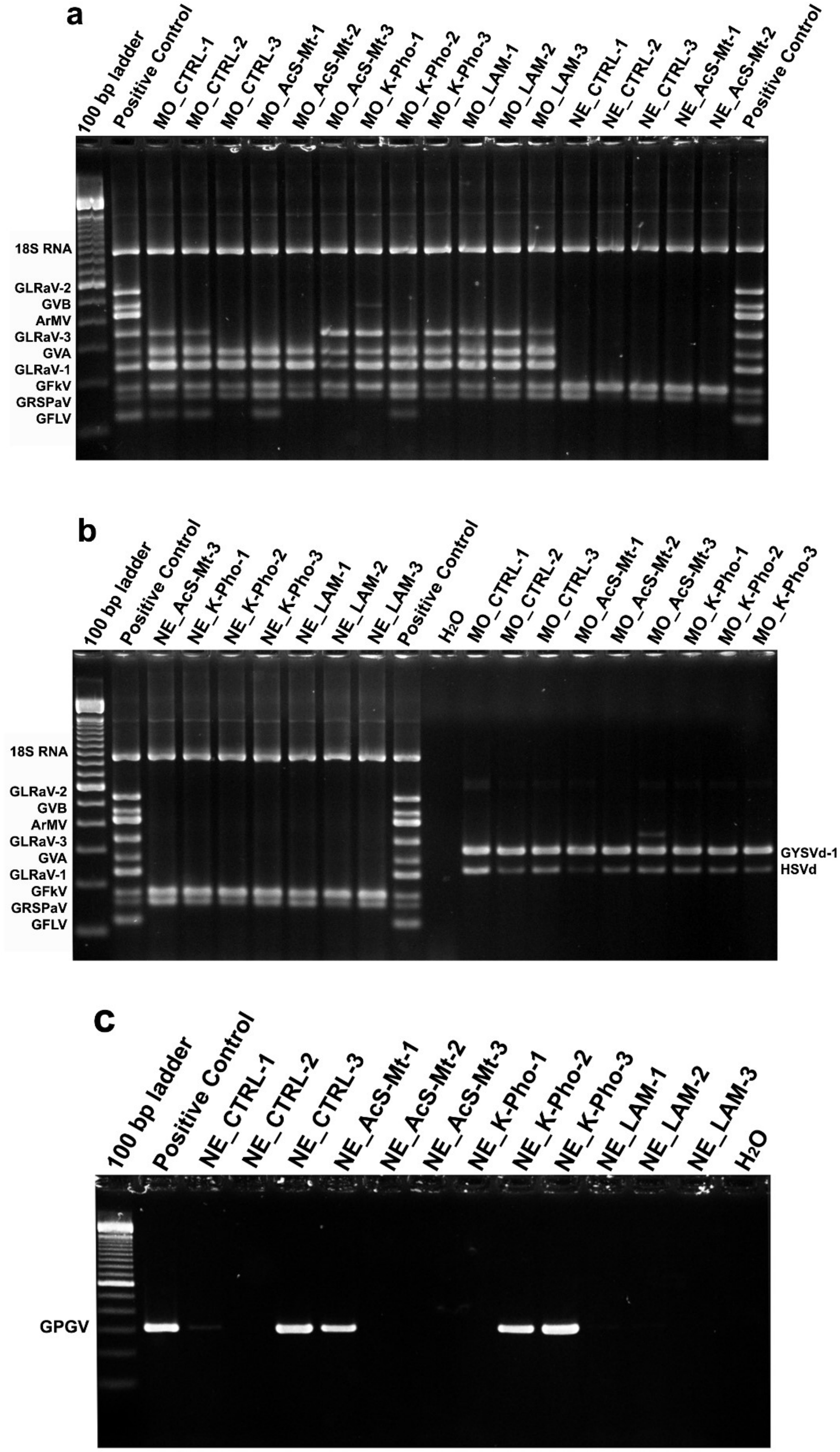

Supplement: Supplementary file 1 [file microorganisms-07-00662-s001.pdf]
